# Frequent problems and their resolutions by using thermal asymmetric interlaced PCR (TAIL-PCR) to clone genes in *Arabidopsis* T-DNA tagged mutants

**DOI:** 10.1080/13102818.2014.998161

**Published:** 2015-01-14

**Authors:** Lei Wu, Dong-Wei Di, Dan Zhang, Bin Song, Pan Luo, Guang-Qin Guo

**Affiliations:** ^a^ Department of MOE Key Laboratory of Cell Activities and Stress Adaptations, School of Life Sciences, Lanzhou University, Lanzhou, Gansu, P.R. China; ^b^ Department of Life Science College, Xiamen University, Xiamen, Fujian, P.R. China

**Keywords:** TAIL-PCR, T-DNA tagged mutants, complex T-DNA insertion, high-throughput sequencing

## Abstract

T-DNA insertional mutagenesis is a powerful tool in *Arabidopsis* functional genomics research. Previous studies have developed thermal asymmetric interlaced polymerase chain reaction (TAIL-PCR) as an efficient strategy in isolation of DNA sequences adjacent to known sequences in T-DNA tagged mutants. However, a number of problems are encountered when attempts are made to clone flanking sequences in T-DNA tagged mutants. Therefore, it is necessary to improve the efficiency of cloning mutagenesis. Here, we present the most frequent problems and provide an improved method to increase TAIL-PCR efficiency. Even then, it is not always possible to successfully obtain flanking sequences; in such cases, we recommend using high-throughput sequencing to determine the mutations.

## Introduction

Over the past years, different strategies have been developed to obtain mutant pools in plants, such as physical or chemical mutagenesis, homologous recombination and transposable element or T-DNA insertional mutagenesis. However, each method has its own characteristics. By physical or chemical mutagenesis, it is easier to get saturated mutant banks, but the mapping of mutation sites is labour intensive and time consuming. The frequency of homologous recombination in plants may be too low to cover the entire genome span.[1]

Thus, as a classic mutagenesis method, *Agrobacterium tumefaciens*-mediated transformation is probably the most widely used method to introduce genes into plants.[2,3] Tumour-inducing plasmid encodes most of the major functions required for transferring an oncogenic segment of DNA, the transferred DNA (T-DNA), into the host cell.[4,5] The T-DNA itself does not include genes required for this transfer process. The distribution of T-DNA insertion sites in the genomes of transgenic plants is random, so transfer of T-DNA mediated by *Agrobacterium* can be highly efficient in plants and can be used to create mutations (for review, see [6]). To date, in the model plant *Arabidopsis thaliana*, whose whole genome sequence is known,[7] there is a large number of T-DNA tagged mutants created by laboratories all over the world, such as SALK,[8] GABI-Kat [9] and Weigel [10]. All of these pools represent near saturation of the entire *Arabidopsis* gene space with mutations and have been widely used for forward and reverse genetic research. Researchers can easily obtain flanking sequences and align with genome database to find mutations.

In our research, we built a forward genetic screen system to isolate mutants from some of the T-DNA tagged mutant pools from the Arabidopsis Biological Resource Center (ABRC) or the Institute of Genetics and Developmental Biology (IGDB) of the Chinese Academy of Sciences (CAS). After obtaining mutants, thermal asymmetric interlaced polymerase chain reaction (TAIL-PCR) [11,12] was used to isolate known DNA sequences adjacent to T-DNA insertions. Unfortunately, we cannot always obtain flanking sequences of T-DNA insertions by TAIL-PCR. In the present study, we develop a more efficient strategy by using a nested three-step TAIL-PCR procedure, 22 short arbitrary degenerate (AD) primers, some specific nested primers of different T-DNA borders and some commercial reagents to clone the mutations. We subjected those mutants for which we failed to obtain T-DNA insertion-flanking sequences by TAIL-PCR, to high-throughput sequencing followed by comparative analysis to identify T-DNA insertion(s).

## Materials and methods

### Mutant pools, vectors, reagents, plant growth and medium

The T-DNA tagged mutant pools CS76502/4/6/8 (PROK2) (ABRC),[8] CS31100 (pSKI015) (ABRC) [10] and DS insertion pools (PWS31) [13] ( IGDB of the CAS) were used to screen mutants. We cloned the flanking sequences in these isolated T-DNA tagged mutants by TAIL-PCR. The DNA polymerase (*TaKaRa rTaq*™) and dNTP were purchased from TaKaRa (Japan). Other reagents were of analytical grade and commercially available. *Arabidopsis* seeds were surface-sterilized by washing in a 20% sodium hypochlorite solution for 10 min, rinsed five times with sterile water, spread on Murashige and Skoog medium [14] with 0.8% agar and grown in a growth chamber at 23 °C.

### Flanking sequence cloning and primers

*Arabidopsis* genomic DNA was extracted by the CTAB (cetyltrimethylammonium bromide) method.[15] CTAB was ordered from Sangon Biotech (China). The flanking sequences of T-DNA insertion were obtained by TAIL-PCR.[11] The primers used for TAIL-PCR are listed in Tables 1 and 2. The settings for TAIL-PCR are shown in (Table 3). All primers were synthesized by Sangon Biotech (China). All PCR products were electrophoresed in a 1% agarose gel, the TAIL-2 and TAIL-3 products showing expected sizes were chosen. TAIL-3 products were purified and sequenced with the chain termination method [16] by Sangon Biotech (China).

**Table 1.** AD primers used in this study.

Primers	Primer sequences (5′–3′)	Tm (°C)	GC (%)	Degeneracy
AD1	NTCGASTWTSGWGTT	39.9	40.00%–46.70%	64
AD2	NGTCGASWGANAWGAA	43.86	37.50%–50.00%	128
AD3	WGTGNAGWANCANAGA	43.86	31.30%–50.00%	256
AD4	WGGWANCWGAWANGCA	48.99	37.50%–50.00%	256
AD5	WCGWWGAWCANGNCGA	51.55	43.80%–56.30%	256
AD6	WGCNAGTNAGWANAA	44	26.70%–46.70%	256
AD7	AWGCANGNCWGANATA	47.71	31.30%–50.00%	256
AD8	SSTGGSTANATWATWCT	48.56	35.30%–41.20%	128
AD9	CGSATSTCSAANAAWAT	48.56	35.30%–41.20%	64
AD10	AGWGNAGWANCAWAGG	46.43	37.50%–50.00%	128
AD11	TGWGNAGWANCASAGA	48.99	37.50%–50.00%	128
AD12	STTGNTASTNCTNTGC	50.27	37.50%–56.30%	256
AD13	CAWCGNCNGANASGAA	52.83	43.80%–62.50%	256
AD14	TCSTNCGNACNTWGGA	52.83	43.80%–62.50%	256
AD15	WCAGNTGWTNGTNCTG	50.27	37.50%–56.30%	256
AD16	TCTTNCGNACNTNGGA	51.55	37.50%–62.50%	256
AD17	TTGNAGNACNANAGG	48.1	33.30%–60.00%	256
AD18	GTNCGASWCANAWGTT	48.99	37.50%–50.00%	128
AD19	NTCAGSTWTSGWGWT	46.73	40.00%–46.70%	128
AD20	TCNGSATWTGSWTGT	46.73	40.00%–46.70%	64
AD21	NCASGAWAGNCSWCAA	51.55	43.80%–56.30%	256
AD22	NTSGASNTCNGAATCA	50.27	37.50%–56.30%	256

**Table 2.** Nested specific primers used in this study.

Primers	Primer sequences (5′–3′)	Tm (°C)	GC (%)	Vector
LBa1	TGGTTCACGTAGTGGGCCATCG	63.8	59.10%	PROK2
LBb1.3	ATTTTGCCGATTTCGGAAC	53.25	42.10%	PROK2
LBb1	GCGTGGACCGCTTGCTGCAACT	65.66	63.60%	PROK2
				
RB1	GTCTGTTGTGCCCAGTCATAG	59.97	52.40%	PROK2
RB1.5	GACCTTAGGCGACTTTTGAACG	60.07	50.00%	PROK2
RB2	ACGGCTTGTCCCGCGTCATC	63.95	65.00%	PROK2
RB3	TGTCGTTTCCCGCCTTCAGT	59.85	55.00%	PROK2
RB4	ATTGGCGGGTAAACCTAAGAGA	58.21	45.50%	PROK2
RB5	GAGAAAAGAGCGTTTATTAGAA	52.62	31.80%	PROK2
				
LB0	TTCTCATCTAAGCCCCCATTTGGAC	61.98	48.00%	pSKI015
LB1	ATACGACGGATCGTAATTTGTC	56.35	40.90%	pSKI015
LB2	TAATAACGCTGCGGACATCTAC	58.21	45.50%	pSKI015
LB3	ACCATCATACTCATTGCTGATCC	58.4	43.50%	pSKI015
LB3'	TTGACCATCATACTCATTGCTG	56.35	40.90%	pSKI015
				
ds5-0.5	GAGAGAGGCAGAGCAGCGTTC	63.9	61.90%	PWS31
ds5-0.6	GGTTATGGATGGGAGTTGGAG	60	52.40%	PWS31
ds5-1	ACGGTCGGGAAACTAGCTCTAC	61.9	54.50%	PWS31
ds5-2	CCGTTTTTGTATATCCCGTTTCCGA	60.3	44.00%	PWS31
ds5-3	TACCTCGGGTTCGAAATCGAT	58	47.60%	PWS31
ds5-4	TAGCATAACGGTACGGTACGG	60	52.40%	PWS31
				
ds3-1	ACCCGACCGGATCGTATCGGT	63.87	61.90%	PWS31
ds3-2	CGATTACCGTATTTATCCCGTTC	58.4	43.50%	PWS31
ds3-3	GTATTTATCCCGTTCGTTTTCGT	56.6	39.10%	PWS31
ds3-4	CCGTCCCGCAAGTTAAATATG	58	47.60%	PWS31

**Table 3.** Settings for TAIL-PCR.

			Reaction system^c^	
Steps	Cycles	Thermal programs	Components	Volume (μL)
TAIL-1	1	95 °C, 2 min	ddH_2_O	32.5
	5	94 °C, 30 s; 67 °C, 1 min; 72 °C, 2.5 min	10 × PCR buffer	5
	1	94 °C, 30 s; 25 °C, 3 min; ramping to 72 °C, 0.3 °C/s; 72 °C, 2.5 min	dNTP (2.5 mmol/L)	4
			Nested specific primer 1 (10 μmol/L)^b^	1
	15	94 °C, 10 s; 67 °C, 1 min; 72 °C, 2.5 min	rTaq™ (5 U/μL)	0.5
		94 °C, 10 s; 67 °C, 1 min; 72 °C, 2.5 min	Template (10–20 ng/μL)	2
		94 °C, 10 s; 44 °C, 1 min; 72 °C, 2.5 min	ADX primer (10 μmol/L)^a^	5
	1	72 °C, 5 min	Total	50
				
TAIL-2	1	95 °C, 2 min	The same as the primary PCR reaction except for:	47
	15	94 °C, 10 s; 64 °C, 1 min; 72 °C, 2.5 min	Nested specific primer 2 (10 μmol/L)^b^	1
		94 °C, 10 s; 64 °C, 1 min; 72 °C, 2.5 min	Template (100-fold diluted TAIL-1 PCR product)	2
		94 °C, 10 s; 44 °C, 1 min; 72 °C, 2.5 min	Total	50
	1	72 °C, 5 min		
				
TAIL-3^d^	1	95 °C, 2 min	The same as the primary PCR reaction except for:	47
	20	94 °C, 15 s; 44 °C, 1 min; 72 °C, 2.5 min	Nested specific primer 3 (10 μmol/L)^b^	1
	1	72 °C, 5 min	Template (10-fold diluted TAIL-2 PCR product)	2
			Total	50

a ADX represent one of the 22 AD primers in Table 1.

b Nested specific primers in Table 2.

c To reduce cost, the reaction system can be appropriately reduced.

d TAIL-3 PCR products were sequenced.

### Sequence analysis

DNA sequence alignments were conducted with the blastn program [17] and tair10 program.[18] High-throughput sequencing technology analyses were provided by ShangHai Biotechnology Corporation (China).

## Results and discussion

### Flanking sequences analysis

TAIL-PCR commonly contains three nested amplifications. The primers used in each amplification reaction consist of left or right border primer, corresponding to the border sequence of the T-DNA, and an AD primer (Table 1). By sequencing a great number of TAIL-3 products, we found that these sequences can be divided into four categories according to their similarity with the plant genome or the vector: (1) Approximately 39% of the DNA fragments whose sequences were as expected, containing several dozens of base pairs homologous to the T-DNA border, and the remainder having significant similarity (E-value < 10^−5^) with the *Arabidopsis* genomic sequence, and thus could be mapped in the *Arabidopsis* genome. (2) Approximately 20% of the DNA fragments had significant similarity only with the vector sequences (either T-DNA or vector backbone). (3) Approximately 13% of the DNA fragments had significantly similar sequences with the *Arabidopsis* genomic sequences, but had no significant similarity with the vector sequences. (4) Approximately 28% had similarity only with the T-DNA border. The lengths of these TAIL-PCR DNA fragments were generally less than 200 bp.

In our experiments, it was difficult to obtain a TAIL-3 DNA fragment in about 8% of the mutants, whereas one or more TAIL-3 fragments were generally generated in the remaining 92% of the mutants. With some exceptions, these DNA fragments contain flanking sequence information as expected. Moreover, some mutants harbouring abnormal T-DNA insertions, such as deleted T-DNA border, tandem repeats and multiple copies of insertion, also make the amplification of the T-DNA insertion-flanking sequences difficult.

### Selected AD primers

To achieve adequate thermal asymmetric priming for TAIL-PCR, the *T_m_*'s (melting temperature) of the AD primers should be at least 10 °C lower than the average *T_m_*'s of the specific primers.[11] Apart from *T_m_*, the factors determining the suitability of an AD primer may include its degeneracy level, length and nucleotide sequence. In previous studies, 15–17 bp length and 64-, 128- or 256-fold AD primers were used for TAIL-PCR. The degeneracy of the arbitrary primers can be created either through inclusion of multiple bases at one position or through inosine incorporation.[11]

Overly high degeneracy levels in AD primers may lead to problems in control of priming efficiency, production of undesirably short DNA fragments and generation of primer--dimer artefacts.[11] However, the low levels of degeneracy in primers is always associated with a decrease in the efficiency of TAIL-PCR to obtain expected DNA fragments. Only when there exist one or more AD primer-binding sites at the *Arabidopsis* genomic sequence which is near the T-DNA border, may the expected specific TAIL-PCR DNA fragments be obtained.

**Figure 1.**
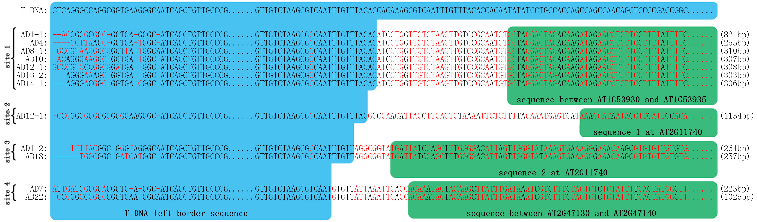
Four T-DNA insertion sites were cloned by TAIL-PCR in the *v*1 mutant. AD1-1 and AD1-2 denote two individual DNA fragments amplified by using AD1. Brackets indicate the length of the flanking sequence.

We used 22 AD primers (Table 1) in our work. As shown in Figure 1, we obtained TAIL-3 products in the *v*1 mutant (screened from CS76502/4/6/8 (PROK2)), followed by sequencing and comparison. Here, we only show all product sequences that were as expected, containing several dozens of base pairs homologous to the T-DNA left border with the remaining sequence having significant similarity (E-value < 10^−5^) with the *Arabidopsis* genomic sequence (Figure 1). The sequence alignment of these products suggests four individual T-DNA insertion sites in the *v*1 mutant.

Although we cloned identical T-DNA insertion sites by using different AD primers, such as the T-DNA insertion site 1 between AT1g53930-AT1g53935 cloned by using AD1, 4, 8, 10, 12, 13 and 14 in the *v*1 mutant (Figure 1), some sites were cloned merely by using a single AD primer, such as the T-DNA insertion site 2 at AT2g11740, which was only detected when the AD12 primer was used (Figure 1). Therefore, using more AD primers can indeed improve the efficiency of TAIL-PCR.

### The T-DNA border deletion phenomena

**Figure 2.**
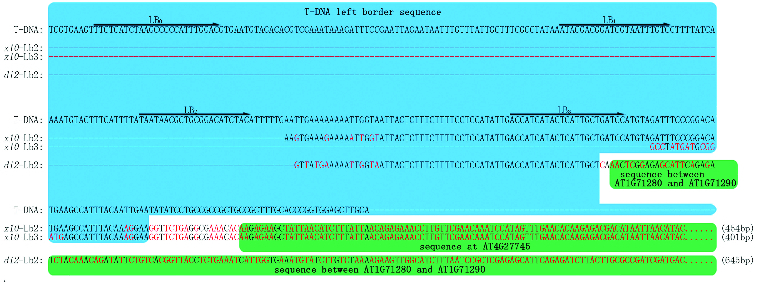
Flanking sequences cloned by TAIL-PCR. Arrows represent the position of the nested specific primers in the T-DNA border. Brackets show the length of flanking sequence. ×10-Lb2 represents the flanking sequences cloned by using three nested primers Lb0, Lb1 and Lb2 in the ×10 mutant. ×10-Lb3 represents the sequences cloned by using Lb1, Lb2 and Lb3 in the ×10 mutant. *di*2-Lb2 represents the sequences cloned by using Lb0, Lb1 and Lb2 in the *di*2 mutant.

According to our sequencing results, approximately 13% of the TAIL-3 DNA fragments only shared significant similarity with the *Arabidopsis* genomic sequence, whereas no obvious similarity with the vector sequence. For instance, the TAIL-PCR product, which was amplified in the ×10 mutant (screened from CS31100 (pSKI015)), only shared similarity (E-value < 10^−5^) with the *Arabidopsis* genomic sequence (Figure 2, product ×10-Lb3). As shown in Figure 2, only 12 bp of the sequence was found to be the same as the T-DNA border sequence. This is too short for the NCBI blast tool to find similar sequences. Therefore, we subsequently designed a new primer Lb0, which is an inner sequence of Lb1, and used the nested primers Lb0, Lb1 and Lb2 instead of Lb1, Lb2 and Lb3 to clone flanking sequences in the ×10 mutant. Interestingly, we obtained the expected DNA fragment (Figure 2, product ×10-Lb2) which contained the T-DNA border sequence and *Arabidopsis* genomic sequence.

In our study, we found that T-DNA insertion can cause a deletion of a different length of its border; importantly, when the border deletion was beyond the primer-binding site, we could not obtain the PCR products. For the *di*2 mutant (screened from CS31100 (pSKI015)), in which we obtained nothing when using Lb1, Lb2 and Lb3, the expected product was obtained by using Lb0, Lb1 and Lb2 (Figure 2, product *di*2--Lb2).

### Reasons for no flanking sequences being amplified and its solution

There exist some complex situations for T-DNA insertion in transgenic plants (for review, see [6]), such as: multiple copies of T-DNA insertions, transfer of vector backbone, complex arrangement of T-DNA, chromosomal duplication and rearrangements or a combination of these.[19–21] Thus, it may be difficult to obtain an expected DNA sequence by TAIL-PCR due to the above-mentioned variable types of insertion behaviour. Here, we summarize some examples derived from our studies.

**Figure 3.**
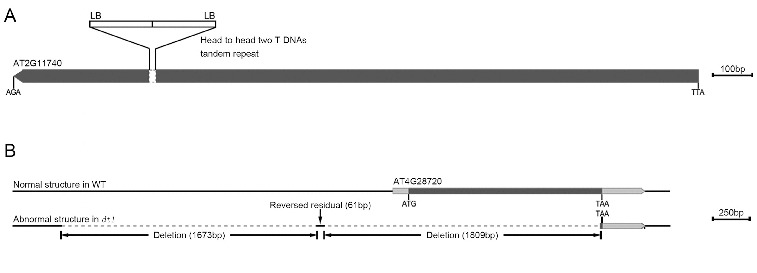
Head-to-head insertion mode in the *v*1 mutant (**A**). Abnormal gene structure in the *dt*1 mutant (**B**).

First, it is often that complex T-DNA insertions composed of two or more T-DNA repeats may be found in transgenic lines.[22] As shown in Figure 1, two T-DNA insertion sites were cloned in the same gene (AT2G11740) in the *v*1 mutant. The sequence analysis shows that these two T-DNAs insert the AT2G11740 gene in a head-to-head orientation. Therefore, we obtained two flanking sequences by using nested left-border specific primers (Figure 3(A)). On the contrary, no flanking sequences were amplified by using right-border primers.

Briefly, we may obtain no flanking sequences when using nested primers from either of the T-DNA borders which is not directly linked with the plant genome, e.g. in a head-to-head orientation. To solve the problem, we designed two sets of nested primers on both the left and the right borders to clone the flanking sequences. However, for some T-DNA borders which contain multiple repeats, it is difficult to design primers (for example, the T-DNA right border of the pSKI015 vector contains 4 × 35S promoters); we can only use nested primers in one border to clone flanking sequences.

Second, in transgenic plants, there are some multiple tandem T-DNA arrays. Some of them show truncated T-DNA regions, some T-DNA regions beyond the border, even sequences of the vector which are far beyond the defined T-DNA region, when the T-DNAs are transferred into the plant genome.[6] In our work, we have observed this to result in a lot of TAIL-PCR product sequences only containing the vector sequence, rather than the plant genomic sequence.

Third, we were not able to amplify the flanking sequence in a few mutants, such as the *dt*1 mutant (screened from CS31100 (pSKI015)). In order to clone the mutation, we used high-throughput sequencing to analyse the *dt*1 genomic sequence. Consequently, we found a change in the gene structure in the vicinity of the AT4G28720 locus (Figure 3(B)). The alignment showed deletions of two DNA segments (1673 and 1809 bp) and a reversed residual of a short segment (61 bp) upstream from the AT4G28720 gene (Figure 3(B)).

By using high-throughput sequencing technology, we have been able to easily obtain a vast amount of genomic sequence information to identify mutations. Nevertheless, there are still small areas of genome sequence which cannot be covered. In our research, in some gametophytic mutants, the mutant gene was heterozygous in the sporophyte. The sequence data from the sporophytic DNA can interfere with the analysis and mutations cannot be effectively identified unless the coverage of high-throughput sequencing data increases. In addition, parts of T-DNA tagged mutations were not linked with the mutant phenotypes that we focused on. To find extra mutations, high-throughput sequencing was also used.

By contrast, for T-DNA tagged mutant pools, the TAIL-PCR method is easier, cheaper and extensively used for identifying mutations in large numbers of mutant samples, while high-throughput sequencing is a more suitable method for identification of those mutations which cannot be identified by using TAIL-PCR.

### Deletion of two or more genes when T-DNAs are inserted into the genome

**Figure 4.**
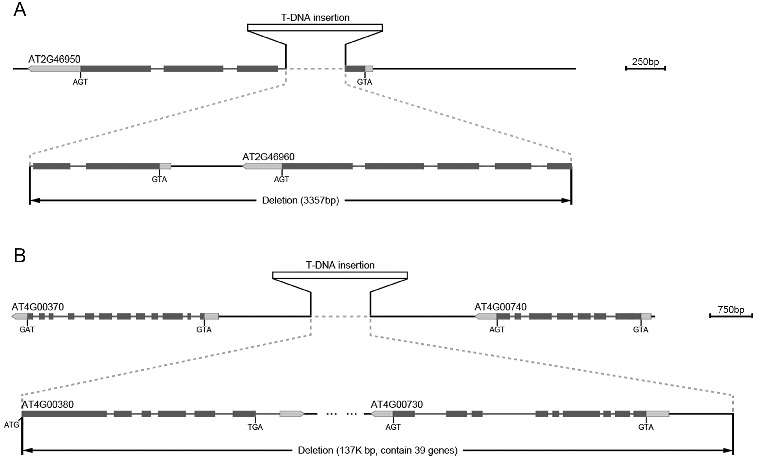
Partial deletion of two genes in the *w*52 mutant (**A**). Deletion of 39 genes in the *kd*361 mutant (**B**).

Using TAIL-PCR, a large number of mutation sites were identified in our work. Among these, there are a few sites whose flanking sequences are very close to each other, such as the distance of 3357 bp in the *w*52 mutant (screened from CS31100 (pSKI015)) (Figure 4(A)) and 137 000 bp in the *kd*361 mutant (screened from DS mutant pools (PWS31)) (Figure 4(B)). The probability of two T-DNA insertions to occur at such a short distance from each other is very low. In the analysis of the *Arabidopsis* genome sequence between these, we found that there was a deletion of the plant genome sequence between the two cloned flanking sequences from one T-DNA insertion (Figure 4 (A and B)).

T-DNA insertion can cause the deletion of two or more genes. For example, there was partial deletion of two genes in the *w*52 mutant (Figure 4(A)) and there were 39 genes deleted in the *kd*361 mutant (Figure 4(B)). Among the studied mutant clones, those with two or more deleted genes were shown to be different from clones bearing single gene mutations. In order to identify the mutants with two or more genes deleted, after completing the analysis of the TAIL-PCR sequences, we always determined the sequence near the two sides of the T-DNA insertion.

## Conclusions

In our studies, for identification of the mutation in each mutant, we first used TAIL-PCR to clone T-DNA flanking sequences and checked whether these T-DNA insertional mutations were linked with phenotypes or not. By using our improved TAIL-PCR method, flanking sequences were cloned in approximately 69% of the mutants. Nevertheless, it was not possible to clone the flanking sequences in the remaining 31% of the mutants (not amplifying TAIL-3 products (8%) and TAIL-3 products containing no flanking sequence information (23%)). Second, the known genes which can cause the same phenotypes need to be checked by allele hybridization and semi-quantitative PCR or real-time quantitative PCR (T-DNA insertional mutagenesis usually causes gene knockout). Finally, after exclusion of the mutations in known possible alleles, high-throughput sequencing was used to find extra mutations. After this, there still needs to be identified which mutation was linked with the phenotypes.
